# Meeting Sustainable Development Goals: Alternative Extraction Processes for Fucoxanthin in Algae

**DOI:** 10.3389/fbioe.2020.546067

**Published:** 2021-01-04

**Authors:** Su Chern Foo, Kuan Shiong Khoo, Chien Wei Ooi, Pau Loke Show, Nicholas M. H. Khong, Fatimah Md. Yusoff

**Affiliations:** ^1^School of Science, Monash University Malaysia, Subang Jaya, Malaysia; ^2^Department of Chemical and Environmental Engineering, Faculty of Science and Engineering, University of Nottingham Malaysia, Semenyih, Malaysia; ^3^School of Engineering, Monash University Malaysia, Subang Jaya, Malaysia; ^4^School of Pharmacy, Monash University Malaysia, Subang Jaya, Malaysia; ^5^Department of Aquaculture, Faculty of Agriculture, Universiti Putra Malaysia, Selangor, Malaysia; ^6^International Institute of Aquaculture and Aquatic Sciences (I-AQUAS), Universiti Putra Malaysia, Port Dickson, Malaysia

**Keywords:** carotenoids, fucoxanthin, algae, health benefits, solvent extraction, alternative extraction processes, sustainability, environmentally friendly

## Abstract

The ever-expanding human population puts tremendous pressure on global food security. With climate change threats lowering crop productivity and food nutritional quality, it is important to search for alternative and sustainable food sources. Microalgae are a promising carbon-neutral biomass with fast growth rate and do not compete with terrestrial crops for land use. More so, microalgae synthesize exclusive marine carotenoids shown to not only exert antioxidant activities but also anti-cancer properties. Unfortunately, the conventional method for fucoxanthin extraction is mainly based on solvent extraction, which is cheap but less environmentally friendly. With the emergence of greener extraction techniques, the extraction of fucoxanthin could adopt these strategies aligned to UN Sustainable Development Goals (SDGs). This is a timely review with a focus on existing fucoxanthin extraction processes, complemented with future outlook on the potential and limitations in alternative fucoxanthin extraction technologies. This review will serve as an important guide to the sustainable and environmentally friendly extraction of fucoxanthin and other carotenoids including but not limited to astaxanthin, lutein or zeaxanthin. This is aligned to the SDGs wherein it is envisaged that this review becomes an antecedent to further research work in extract standardization with the goal of meeting quality control and quality assurance benchmarks for future commercialization purposes.

## The Need for Microalgae to Meet Sustainable Development Goals

A rich biodiversity at the hierarchical levels of ecosystem, species, and genetics (Mace et al., 2012) is the key to sustainable development of humanity (Toledo and Burlingame, 2006). Microalgae represent untapped resources with its biodiversity ranging from 200,000 species to several million species (Norton et al., 1996), as compared to only about 250,000 species in higher plants (Pulz and Gross, 2004). Microalgae provide crucial ecosystem services under their roles in provision (as raw materials for consumption and use), support (as primary producers in aquatic ecosystems), regulation (supplying more than 80% of world oxygen), and food culture (e.g., spirulina was a food source for the Aztecs until the 16th century). The rich taxonomic diversity of microalgae is attractive not only for bioprospecting but also to produce valuable and diverse bioactive compounds such as carotenoids, proteins, enzymes, polyunsaturated fatty acids (PUFAs), lipids, or exopolysaccharides (EPS). Microalgae are photosynthetic and can be found in extreme ecosystems including polar regions, hot springs, deserts, ocean, and micro-aerobic environments. The versatility of microalgae makes them very promising candidates as crops for the future. Combining the latest cultivation and harvesting technologies for improvements in resource efficiency, microalgae could be a new impetus to the current lag in global economic growth.

Sustainable development goals (SDGs; 2015–2030), an extension of millennium development goals (MDGs; 2000–2015), were adopted by all United Nations Member States as a universal call to action for poverty eradication, global prosperity and, protection of the environment (Sachs, 2012). Microalgae, as a carbon-neutral resource, have directly contributed to three out of the eight MDGs (i.e., reducing poverty and hunger, promoting environmental sustainability, and developing global partnerships). Further to this, the application of microalgae in biotechnological platforms could contribute to sixteen out of the seventeen SDGs, detailed in Table 1.

**TABLE 1:** Summary of the roles of microalgae in achieving SDGs.

**SDG**	**Main targets**	**Microalgae applications**	**References**
(1) No poverty	Wealth creation	Aside from using terrestrial biomass like barley or hop, alternative aquatic biomass from *Chlorella vulgaris* could be opted for energy and revenue creation	Kusin and Horan, 2015
(2) Zero hunger	• End hunger • Achieve food security and improved nutrition • Promote sustainable agriculture	Microalgae is a cell factory for essential nutrients to support human health. Nutrients from microalgae has been used in the past as a diet supplement for undernourished children. Whereas microalgae by-products can be used as feed additives in poultry and aquaculture	Matondo et al., 2016; García et al., 2017; Kwon et al., 2019
(3) Good health and well being	• Ensure healthy lives • Promote well-being for all at all ages	Microalgae extracts are found to be effective in preventing and treating both communicable and non-communicable diseases	Yan et al., 2016; Foo et al., 2017, 2019; Kawee-Ai et al., 2019
(4) Quality education	• Ensure inclusive and equitable quality education for all • Promote life-long learning opportunities	The availability of microalgae in culture collections enables for worldwide accessibility in different forms of teaching, research, or industrial training. This in turn promotes an inclusive and equitable education to all	Blackburn et al., 1997, 2005
(5) Gender equality	Gender equality and empowerment of women and girls	Empowerment and scholarship opportunity for women in science, technology, engineering, and mathematics (STEM) industries especially for those working in the fields of agricultural and blue biotechnology involving microalgae	Jelić and Jovanović, 2013
(6) Clean water and sanitation	Ensure availability and sustainable management of water and sanitation for all	Microalgae-bacteria symbiosis improves water quality by removing organic matter, excessive nutrients, hazardous contaminants and heavy metals	Abdel-Raouf et al., 2012; Kumar et al., 2015
(7) Affordable and clean energy	Ensure access to affordable, reliable, sustainable, and modern energy for all	Microalgae are the fastest growing aquatic plant and they have the potential to yield more oil or biomass per ha when compared to terrestrial crops and plants. For example, hydrocarbons from *Botryococcus braunii* can replace fossil fuels	Banerjee et al., 2002; Bayro-Kaiser and Nelson, 2017; Moriarty and Honnery, 2019
(8) Decent work and economic growth	Promote sustained, inclusive, and sustainable economic growth, full and productive employment, and decent work for all	Microalgae can improve sustainability practices of existing industrial activities, including wastewater treatment and the production of pharmaceuticals, cosmetics, feed, food, and biofuel	Simas-Rodrigues et al., 2015; Richmond, 2017
(9) Industry, innovation and infrastructure	Build resilient infrastructure, promote inclusive and sustainable industrialization and foster innovation	Microalgae are used as an innovative greening component in future buildings. For example, microalgae were cultured in photobioreactors that were retrofitted or architecturally planned as bio-facades of buildings to harvest solar energy for electricity conversion	Mohseniazar et al., 2011; Qiu, 2014; Dahoumane et al., 2016; Elrayies, 2018; Kashyap et al., 2019
(10) Reduced inequalities	Reduce inequality within and among countries	More development interventions (e.g., equal access to credit and information for women) should be implemented for example, development policies like “Kenya Vision 2030” with the goal of using algae biomass as a source of food, feed and biofuel for the country, recognizes the role of women in policy implementation	Derun, 2009; Moejes and Moejes, 2017
(11) Sustainable cities and communities	Make cities and human settlements inclusive, safe, resilient, and sustainable	Sustainable architecture is one of the approaches to energy conservation in view of the increasing human population. A cost-benefit analysis from an environmental perspective showed that the closed tubular microalgae photobioreactor system has more benefits as compared to solar panel system	Biloria and Thakkar, 2020; Talebi et al., 2020
(12) Responsible consumption and production	Ensure sustainable consumption and production patterns	A circular approach was demonstrated using *Spirulina* as a bio-template in the synthesis of photocatalysts for water decontamination, and at the same time, the remaining biomass was used for the bioethanol production. Similarly, microalgae-based biorefinery can promote circular economy based on techno-economic and life-cycle analyses	Kavitha and Gunasekaran, 2020; Serrà et al., 2020
(13) Climate action	Take urgent action to combat climate change and its impacts	Microalgae are effective in capturing carbon dioxide, a greenhouse gas that contributes to climate change	Singh et al., 2019
(14) Life below water	Conserve and sustainably use the oceans, seas and marine resources for sustainable development	Microalgae could be a bioindicator for assessing the climate change as well as the terrestrial influences on marine health and ecology of coral reefs. If microalgae were used to replace fishmeal and fish oil globally, the effect would be equivalent to 30% of reduction in fishing pressure at the lower end of the food web; this would contribute to restoration of the marine ecosystem	Blanco et al., 2008; Hemaiswarya et al., 2011; Beal et al., 2018; Gaignard et al., 2019
(15) Life on land	Protect, restore and promote sustainable use of terrestrial ecosystems, sustainably manage forests, combat desertification, and halt and reverse land degradation and halt biodiversity loss	Microalgae are an effective pioneer microorganism in the restoration of acrid soil and the desertification where several successful cases of implementations have been reported	Lababpour, 2016
(16) Peace, justice and strong institution	Promote peaceful and inclusive societies for sustainable development, provide access to justice for all and build effective, accountable and inclusive institutions at all levels	n/a	n/a
(17) Partnerships for the goals	Strengthen the means of implementation and revitalize the global partnership for sustainable development	CyanoFactory is a R&D project focusing on the design and construction of novel photosynthetic cell factories for solar biofuel production. Such examples of “purpose driven” research that are supported by the scientific goals and the need for creation of new technologies requires more interdisciplinary partnerships to promote the application of microalgae to a greater level	Akinsemolu, 2018; Lindblad et al., 2019
			

## The Potential of Fucoxanthin

### Introduction to Fucoxanthin: Structure and Function

**FIGURE 1:**
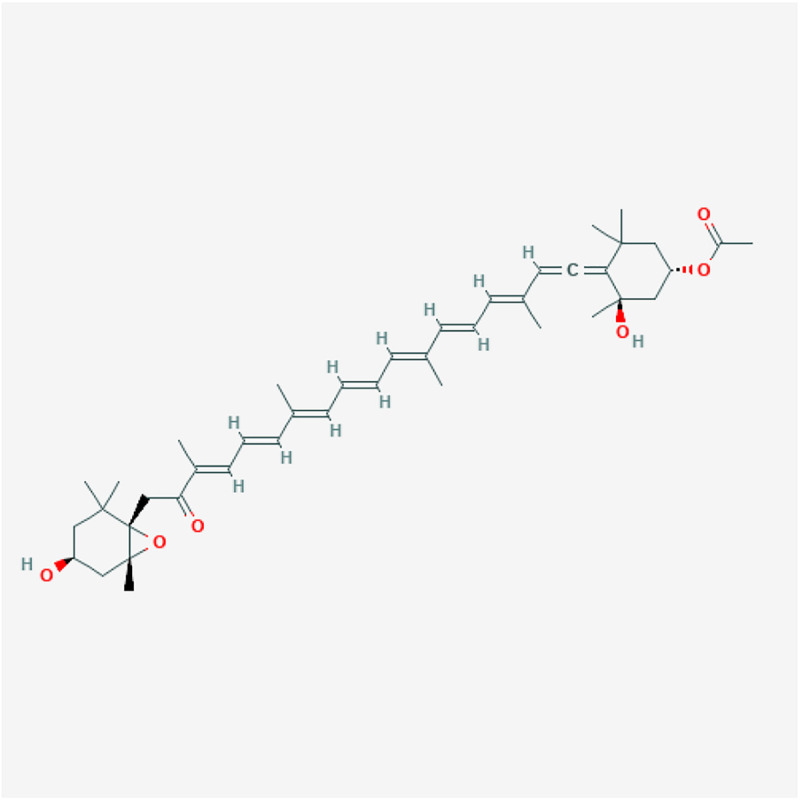
The 2D structure image of CID 5281239 (fucoxanthin). Source: https://pubchem.ncbi.nlm.nih.gov/compound/5281239#section=2D-Structure.

Fucoxanthin is a xanthophyll with an unusual epoxide group, allenic bond and, conjugated carbonyl group on the polyene molecule that sets it apart from the other carotenoids (Peng et al., 2011). The major structure responsible for the antioxidant and light-harvesting properties in carotenoids is the conjugated double-bond system in the chromophore (Figure 1). Carotenoids in microalgae are efficient scavengers for singlet molecular oxygen (^1^O_2_) and peroxyl radicals (ROO) (Stahl and Sies, 2012). In radical scavenging, carotenoids act as a radical trap and add electrons to their conjugated double-bond, yielding a ground-state oxygen and a triplet-state carotenoid. Subsequently, the excited carotenoid structure dissipates the excess energy to the surrounding environment by returning to its ground state (Takashima et al., 2012).

The antioxidant activities of fucoxanthin have been well characterized in the past. Fucoxanthin demonstrated proton-donative activities in the 1,1-diphenyl-2-picrylhydrazyl (DPPH) assay (Nomura et al., 1997). Moreover, fucoxanthin and its metabolites (fucoxanthinol and halocynthiaxanthin) exhibited radical scavenging activity and singlet oxygen quenching activity (Sachindra et al., 2007). Similarly, research in the last few years showed that fucoxanthin not only could scavenging radicals but also was effective in chelating iron (Foo et al., 2015b) and slowing down the bleaching of β-carotene in linoleic acid emulsion systems (Foo et al., 2015a).

### Fucoxanthin Sources

Unlike β-carotene and lutein that are found ubiquitously in terrestrial crops, fucoxanthin is obtained exclusively from the aquatic environment. Fucoxanthin is the signature pigment markers for macroalgae and microalgae species listed in Table 2. This includes Phaeophyceae (e.g., *Laminaria japonica, Sargassum fulvellum, Undaria pinnatifida*), Bacillariophyceae (e.g., *Odontella aurita, Chaetoceros* sp., *Phaeodactylum tricornutum, Cylindrotheca closterium*), Prymnesiophyceae (e.g., *Isochrysis galbana, Pavlova lutheri*), Chrysophycea (e.g., *Pelagococcus subviridis*), Raphidophyceae (e.g., *Psammamonas australis*) and Dinophyceae (e.g., *Kryptoperidinium foliaceum*) (Wright and Jeffrey, 1987; Stauber and Jeffrey, 1988; Bjornland and Liaeen-Jensen, 1989; Grant et al., 2013). The annual production of microalgae biomass in 2000–2004 was at 5000–10000 tons (Van Harmelen and Oonk, 2006). Due to increasing market demand, this number has almost doubled to 20000 tons (Tredici et al., 2016) in the last 10 years or so. Whilst in 2003, the world production of macroalgae or seaweed reached 8 million tons and was valued at US$ 5.5–6 billion (McHugh, 2003). A report by Roesijadi et al. (2010) showed global monetary value of seaweeds to have reached over US$ 7 billion and was projected to continue with increasing human population growth. From here, microalgae and macroalgae biomass are the prospective and sustainable feedstock of the future.

**TABLE 2:** Fucoxanthin in microalgae and macroalgae.

**Type**	**Class**	**Species**	**Fucoxanthin quantity (mg.g^–1^ DW)**	**References**
Microalgae	Bacillariophyceae	*Phaeodactylum tricornutum*	15.42–16.51	Kim et al., 2012
		*Chaetoceros calcitrans*	2.33 ± 0.14	Goiris et al., 2012
		*Chaetoceros calcitrans* (Paulsen) Takano 1968	5.25 ± 0.03	Foo et al., 2015b
		*Phaeodactylum tricornutum* SCSIO828	5.50	Wu et al., 2015
Microalgae	Prymnesiophyceae	*Isochrysis galbana*	7.75 ± 0.13 18.23	Goiris et al., 2012; Kim et al., 2012
		*Pavlova lutheri*	17.9	Griffiths and Harrison, 2009
Microalgae	Chrysophyceae	*Dinobryon cylindrical*	n.d	Withers et al., 1981
		*Ochromonas* sp.	0.03–0.12	Withers et al., 1981
Macroalgae	Phaeophyceae	*Laminaria japonica*	1.85	Xiao et al., 2012
		*Laminaria digitata*	0.468	Holdt and Kraan, 2011
		*Undaria pinnatifida* (Harv.) Sur.	0.727	Xiao et al., 2012
		*Sargassum fusiforme*	0.0133	Xiao et al., 2012
		*Sargassum duplicatum*	1.01 ± 0.10	Noviendri et al., 2011
		*Sargassum binderi*	0.73 ± 0.39	Noviendri et al., 2011
		*Myagropsis myagroides*	9.01	Heo and Jeon, 2009
		*Dictyota coriacea*	6.42	Heo and Jeon, 2009
		*Eisenia bicyclis*	0.109	Airanthi et al., 2011
		*Kjellmaniella crassifolia*	0.152	Airanthi et al., 2011
		*Alaria crassifolia*	0.041	Airanthi et al., 2011
		*Sargassum horneri*	0.020	Airanthi et al., 2011
		*Cystoseira hakodatensis*	0.0041	Airanthi et al., 2011
		*Turbinaria turbinata*	0.59 ± 0.08	Jaswir et al., 2013
		*Sargassum plagiophyllum*	0.71 ± 0.08	Jaswir et al., 2013

There has been an increasing number of scientific reports showing the potential of fucoxanthin to improve the nutritional quality of food, which in turn benefits the health and well-being of consumers. This shows great opportunities for fucoxanthin from both microalgae and macroalgae to be commercialized and made available on food shelves. Intriguingly, the ultimate nutraceutical sources are mainly derived from producers at the beginning of the food chain (Shahidi and Alasalvar, 2010). By careful control of nutrients, a wide spectrum of biochemicals can be synthesized by algae and be used as safe and value-adding ingredients in food and nutraceutical sectors. Not only can algae act as suitable nutrition cell factories to meet food demand, but they could also be a healthy, *halal* and clean plant source for vegetarians and vegans. It would thus be encouraging to make fucoxanthin more accessible to consumers.

### Fucoxanthin Applications

In the last decade, fucoxanthin has found applications as nutraceuticals and more recently in nutricosmetics. Fucoxanthin is classified as a type of nutraceutical because it exerts therapeutic effects (e.g., anti-inflammatory, anti-obesity, anti-diabetic, and anti-cancer) as presented in Table 3. Fucoxanthin can be incorporated into conventional food (e.g., milk, rice, bread, and pasta) as a bioactive ingredient to increase the nutritional value and enhance the sensory qualities (Prabhasankar et al., 2009; Abu-Ghannam and Shannon, 2017). Besides that, fucoxanthin has found application in nutricosmetics as anti-obesity pills or “oral cosmetics” (Agatonovic-Kustrin and Morton, 2013; Couteau and Coiffard, 2016). Fucoxanthin is a good ingredient of nutricosmetics because it has demonstrated slimming effects by inhibiting fat absorption (Muradian et al., 2015).

**TABLE 3:** Therapeutic effect and toxicity studies of fucoxanthin.

**Activity(s)**	**Models**	**Results**	**Mechanism of action**	**References**
Anti-inflammation	RAW 264.7 macrophages	Inhibitory effects on inflammatory cytokines and mediators	Inhibition of nuclear factor-κB gene activation and phosphorylation of mitogen-activated protein kinases	Heo et al., 2012
	Mast cells	Suppression of mast cell degranulation *in vivo*	Suppression of antigen-induced aggregation of high-affinity IgE receptor and activation of degranulating signals of mast cells	Tan and Hou, 2014
Anti-obesity	Wistar rats and KK-Ay mice	A reduction of abdominal white adipose tissue weights in subjects	Inhibition of fat absorption and decreased serum triglyceride level by induction of uncoupling protein-1 (UCP-1)	Maeda et al., 2007
Anti-diabetic	Diabetic/obese mice	Fucoxanthin decreased the blood glucose and plasma insulin levels thereby improving alterations in lipid metabolism and insulin resistance induced by a high-fat diet via reduction of visceral fat mass, hyperinsulinemia, hepatic glucose production, and hepatic lipogenesis	Via downregulation of adipokines like tumor necrosis factor-α, monocyte chemoattractant protein-1, interleukin-6 and, plasminogen activator inhibitor-1	Miyashita et al., 2012
Anti-cancer	MCF-7 breast cancer cell line	*Chaetoceros calcitrans* extracts were able to induce apoptosis at a concentration as low as 3 ppm	Fucoxanthin increased the expression of apoptotic genes, resulting in an increase in BAX/Bcl-2 ratio and activation of caspase 7 mRNA expression	Ebrahimi Nigjeh et al., 2013
	HepG2 cancer cell line	*Chaetoceros calcitrans* extracts induced cytotoxicity to HepG2 cells following concentration and time-dependent pattern	Modulation of numerous genes involved in cell signaling (AKT1, ERK1/2, and JNK), apoptosis (BAX, BID, Bcl-2, APAF, and CYCS), and oxidative stress (SOD1, SOD2, and CAT)	Foo et al., 2019
	BNL CL.2 transformed murine liver cells	Fucoxanthin acted as a cell signaling inhibitor at 12 h incubation (5 μM) of *Undaria pinnatifida* extracts	Fucoxanthin activated the Nrf2/ARE pathway by increasing the expressions of HO-1 and NQO-1 expression primarily through the ERK/P38 pathway	Liu et al., 2011
Anti-aging	Topical application on HOS: HR-1 hairless mice	Suppression of UVB-induced wrinkle formation	Fucoxanthin prevents skin photoaging via antioxidant and antiangiogenic effects	Urikura et al., 2011
Toxicity and safety evaluation	Single and repeated oral dose toxicity studies	No fatalities or abnormalities reported in both studies	Low toxicity and safe for consumption. Normal histology and no abnormal changes in liver, kidney, spleen, and gonadal tissues	Beppu et al., 2009
Multiple drug resistance (MDR)	Caco-2 and CEM/ADR5000 cells	Fucoxanthin exhibited a chemosensitizers role to alleviate MDR	By acting as competitive inhibitors of ATP-binding cassette (ABC)	Batista et al., 2012
Metabolism, bioavailability, and safety	Oral administration to mice	Results demonstrated dietary fucoxanthin accumulates in the heart and liver as fucoxanthinol and in adipose tissue as amarouciaxanthin A	This indicates that the bioavailability of fucoxanthin (and its metabolites) may be higher than that of other xanthophylls, at least of astaxanthin	Hashimoto et al., 2009
	13 groups of C57BL/6J mice	No toxicological effects were observed. Neither histological nor serum analyses revealed any heart, kidney, or liver toxicity induced by algae diets	Algae-rich diets were thus well accepted, well-tolerated and suitable for the maintenance of body weight and normal organ function	Neumann et al., 2018

In the present market, microalgae cosmetic products from carotenoids are mainly extracted from the chlorophycean, *Nannochloropsis oculata* (Shen et al., 2011). As microalgae are considered “superfoods,” they are composed of amino acids, essential fatty acids, vitamins, key minerals, trace elements, antioxidants, electrolytes, nucleic acids and, enzymes that play important roles in cellular regeneration. Urikura et al. (2011) demonstrated the protective effects of fucoxanthin against UVB-induced skin photoaging as evidenced in hairless mice. Compounds responsible for such activity included phlorotannins, polysaccharides, carotenoid pigments (fucoxanthin), and fucosterol in brown algae (Kim, 2011). With more focus on natural carotenoids, there is a huge market of opportunity for the incorporation of these bioactive ingredients to replace synthetic colorings in food and nutricosmetics.

## Fucoxanthin Extraction Processes

The isolation of fucoxanthin from microalgae feedstock can be attained by various extraction methods. The selection of extraction techniques is driven by the cost of operation, the complexity of feedstock, demand for the quality and the yield of final bioproducts. For example, to commercialize fucoxanthin in the fields of pharmaceutical, cosmetics, food or analytical testing, the bioactivity and purity of fucoxanthin must be well preserved. The characteristics of microalgae biomass possess a challenge to the extraction of fucoxanthin (Abu-Ghannam and Shannon, 2017) and one of them is the type of cell wall (e.g., cellulosic or siliceous). Hence, the extraction parameters influencing the performance of extraction need to be identified and optimized for maximizing the product yield while minimizing the operation time, chemical consumption, utility cost and waste generation. In the past, fucoxanthin extraction from microalgae feedstock was achieved by organic-solvent-based extraction with the aid of maceration or Soxhlet extraction. To date, alternative extraction techniques have been adopted as an environmentally friendly route to extract fucoxanthin. In the following sections, the conventional and alternative methods for extraction of fucoxanthin were reviewed and compared in the aspects of working principle, extraction performance, strength, and weakness of the method.

### Conventional Solvent Extraction Methods for Fucoxanthin

The common techniques for extraction of carotenoids include maceration (soaking or direct organic-solvent extraction), Soxhlet extraction, or steam/hydro distillation (Kadam et al., 2013; Khoo et al., 2019). In general, the selection of organic solvent and the operation cost must be taken into consideration (Zarekarizi et al., 2019) when treating different types of macroalgae or microalgae for carotenoid extraction (Table 4). In addition, the operation involving organic solvents must be handled with care because of the highly volatile and flammable characteristics of these solvents. Examples of organic solvents used in the solvent extraction of fucoxanthin are acetone, methanol, ethanol, n-hexane, dimethyl sulfoxide, dichloromethane, tetrahydrofuran and ethyl acetate. The properties of solvent systems, including dielectric constant and polarity index, affect the extraction yield of carotenoids. Fucoxanthin can be dissolved in mid-polar solvent systems because of the semi-polar characteristic of fucoxanthin and the oxygen molecule in the fucoxanthin structure, but water was found to be ineffective in solubilizing fucoxanthin (Aslanbay Guler et al., 2020). Although acetone was commonly used in the direct solvent extraction of fucoxanthin (Abu-Ghannam and Shannon, 2017), the yield of extraction was typically lesser than that by ethanol due to the lack of hydroxyl functional group for better hydrophilic interaction. Tetrahydrofuran was found to be less efficient in extracting fucoxanthin because it generates peroxides that degrade fucoxanthin (Aslanbay Guler et al., 2020). A previous study showed that the ethanolic extraction of fucoxanthin from diatom *P. tricornutum* yielded 15.71 mg/g dried weight (Kim et al., 2012). Similarly, a recent study found that the extraction of fucoxanthin from *P. tricornutum* was governed by the type of solvent used; the selectivity of solvent for fucoxanthin was in the descending order of d-limonene > ethyl acetate > ethyl lactate > ethanol (del Pilar Sánchez-Camargo et al., 2017).

**TABLE 4:** Conventional extraction methods and fucoxanthin yield from microalgae and macroalgae.

**Extraction methods**	**Type of solvent**	**Species**	**Class**	**Temperature (°C)**	**Fucoxanthin yield**	**References**
Solvent extraction or maceration	Ethanol	*Phaeodactylum tricornutum*	Bacillariophyceae	30	15.71 mg/g	Kim et al., 2012
	Ethanol	*Odontella aurita*	Bacillariophyceae	45	17.20 mg/g	Xia et al., 2013
	Ethanol (96%)	*Sargassum muticum*	Phaeophyceae	40	0.55 mg/g	Conde et al., 2014
	Methanol	*Chaetoceros calcitrans*	Bacillariophyceae	25	22.71%	Foo et al., 2015a
	Acetone	*Phaeodactylum tricornutum*	Bacillariophyceae	25	4.60 mg/g	Kim et al., 2012
	Ethyl acetate	*Phaeodactylum tricornutum*	Bacillariophyceae	25	2.26 mg/g	Kim et al., 2012
	Dimethyl sulfoxide	*Laminaria japonica*	Phaeophyceae	25	122.10 μg/g	Wang et al., 2005
	Acetone	*Fucus vesiculosus*	Phaeophyceae	30	0.70 mg/g	Shannon and Abu-Ghannam, 2017
	Tetrahydrofuran	*Phaeodactylum tricornutum*	Bacillariophyceae	35	1.28 mg/g	Aslanbay Guler et al., 2020
	Dichloromethane	*Phaeodactylum tricornutum*	Bacillariophyceae	35	1.28 mg/g	Aslanbay Guler et al., 2020
	Methanol	*Phaeodactylum tricornutum*	Bacillariophyceae	35	0.57 mg/g	Aslanbay Guler et al., 2020
	Acetone + methanol (1:1, v/v)	*Saccharina japonica*	Phaeophyceae	25	0.48 mg/g	Sivagnanam et al., 2015
	Acetone + methanol (1:1, v/v)	*Sargassum horneri*	Phaeophyceae	25	0.71 mg/g	Sivagnanam et al., 2015
	Hexane	*Saccharina japonica*	Phaeophyceae	25	0.16 mg/g	Sivagnanam et al., 2015
	Hexane	*Sargassum horneri*	Phaeophyceae	25	0.05 mg/g	Sivagnanam et al., 2015
	Ethanol	*Saccharina japonica*	Phaeophyceae	25	0.12 mg/g	Sivagnanam et al., 2015
	Ethanol	*Sargassum horneri*	Phaeophyceae	25	0.08 mg/g	Sivagnanam et al., 2015
	Cold acetone-methanol (7:3 v/v)	*Sargassum binderi*	Phaeophyceae	25	0.73 mg/g	Noviendri et al., 2011
	Cold acetone-methanol (7:3 v/v)	*Sargassum duplicatum*	Phaeophyceae	25	1.01 mg/g	Noviendri et al., 2011
	Acetone, 120 min	*Cylindrotheca closterium*	Bacillariophyceae	20	5.34 μg/mg	Pasquet et al., 2011
	Acetone, 60 min	*Cylindrotheca closterium*	Bacillariophyceae	56	5.23 μg/mg	Pasquet et al., 2011
Soxhlet extraction	Ethanol (80%)	*Phaeodactylum tricornutum*	Bacillariophyceae	80	15.42 mg/g	Kim et al., 2012
	Ethanol	*Laminaria japonica*	Phaeophyceae	40	191 μg/g	Kanazawa et al., 2008
	Ethanol	*Undaria pinnatifida*	Phaeophyceae	78	50 μg/g	Kanda et al., 2014

Moreover, the extraction of fucoxanthin from *P. tricornutum* can be improved by the application of a hot soaking process with acetone (Pasquet et al., 2011). However, this approach caused the degradation of chlorophyll *a* from diatom *C. closterium*, while the cold soaking process rendered the chlorophyll *a* to be partially decomposed after 60 min. Similarly, the extraction efficiency of fucoxanthin from *P. tricornutum* was improved when the temperature of ethanol (50%) increased from 30 to 70°C (Kim et al., 2012). Nonetheless, an extremely high temperature condition of solvent extraction (e.g., above the boiling point of solvent) could cause the localized overheating effects on the fucoxanthin that render its degradation and low recovery.

Soxhlet extraction is a solid-liquid extraction approach that involves the continuous mass transfer of non-volatile target compounds via reflux of organic solvents. The efficiency of extraction depends on the selectivity of solvents for the compounds, the diffusion rate of solvents, and the solubility of target compounds in the solvents (Kim, 2012). Although Soxhlet extraction is a simple diffusion process without applying shear stress to the biomass, it is unsuitable for the extraction of temperature-sensitive carotenoids as their bioactivity will be degraded during the heating cycles (Kim et al., 2012).

Although the maximum yield of the product remains a priority, the selection of solvents should thoroughly consider other criteria such as environmental impact, toxicity and sustainability of the selected solvent (Table 4). For instance, in the extraction of fucoxanthin from *P. tricornutum*, the extraction efficiency of methanol was higher than that of ethanol (Aslanbay Guler et al., 2020). However, by considering the toxicity of solvents, methanol is relatively hazardous to both environment and human use. Other alternative organic solvents such as petroleum ether and n-hexane are typically incompatible with the extraction of fucoxanthin because of their hydrophobic properties. Moreover, solvent extraction often suffers from the large consumption of organic solvent (Khoo et al., 2019). Nonetheless, the feasibility of recycling solvents via distillation and evaporation under vacuum could mitigate the chemical consumption and waste generation. More importantly, the extracted fucoxanthin must be depleted of organic solvents used in the solvent extraction process if the final product is used as a functional ingredient in food or supplements.

### Alternative Fucoxanthin Extraction Methods

The importance of SDG no. 12 (i.e., responsible consumption and production) has become more prominent as the world faces challenges in coping with pollution problems and food demand. The sustainability in the food supply chain and the low-carbon footprint of the commercial food products should begin with the growth of sustainable crops as well as the greener extraction methods in processing of bioproducts. The emergence of alternative extraction methods has opened new avenues to the sustainable extraction of fucoxanthin from algal sources. Table 5 shows the emerging methods for extraction of fucoxanthin from macro- and microalgae.

**TABLE 5:** Emerging methods for extraction of fucoxanthin from macro- and microalgae.

**Extraction method**	**Extractive solvents**	**Operating conditions**	**Algae strains**	**Yield of fucoxanthin**	**References**
SFE	SC-CO_2_	70°C, 40 MPa, 3 h	*Undaria pinnatifida* (seaweed)	59.51 μg/g	Kanda et al., 2014
	SC-CO_2_ + ethanol (3.23%)	60°C, 40 MPa, 3 h	*Undaria pinnatifida* (seaweed)	994.53 μg/g	Kanda et al., 2014
	SC-CO_2_	50°C, 30 MPa, 1 h	*Sargassum muticum* (seaweed)	1.5 mg/100 g	Conde et al., 2014
	SC-CO_2_ + ethanol (10%)	50°C, 20 MPa, 40 min	*Sargassum muticum* (seaweed)	12 mg/100 g	Conde et al., 2014
	SC-CO_2_	40°C, 40 MPa, 3 h	*Undaria pinnatifida* (seaweed)	1.22 g/100 g	Quitain et al., 2013
	SC-CO_2_	60°C, 40 MPa, 150 min	*Undaria pinnatifida* (seaweed)	58 μg/g	Goto et al., 2015
	SC-CO_2_	40°C, 30 MPa, 3 h	*Saccharina japonica* (seaweed)	2.08 mg/g	Getachew et al., 2018
	SC-CO_2_ + methanol	30°C, 20 MPa, 1 h	*Phaeodactylum tricornutum* (diatom)	0.69 mg/g	Aslanbay Guler et al., 2020
	SC-CO_2_ + ethanol (10%)	45°C, 25 MPa, 2 h	*Saccharina japonica* (seaweed)	0.41 mg/g	Sivagnanam et al., 2015
	SC-CO_2_ + ethanol (10%)	45°C, 25 MPa, 2 h	*Sargassum horneri* (seaweed)	0.77 mg/g	Sivagnanam et al., 2015
	SC-CO_2_ + ethanol (10%, v/v)	50°C, 20 MPa, 2 h	*Undaria pinnatifida* (seaweed)	0.00753 μg/g	Roh et al., 2008
PLE	Ethanol: water (9:1)	110°C, 10.3 MPa	*Eisenia bicyclis* (brown algae)	0.42 mg/g	Shang et al., 2011
	Ethanol	100°C, 10.3 MPa	*Phaeodactylum tricornutum* (diatom)	16.51 mg/g	Kim et al., 2012
	Dimethyl ether	25°C, 10.3 MPa	*Undaria pinnatifida* (seaweed)	390 μg/g	Kanda et al., 2014
	Ethanol	100°C, 10.3 MPa	*Phaeodactylum tricornutum* (diatom)	7.73 mg/g	Gilbert-López et al., 2017
	Ethanol	170°C, 10.3 MPa	*Phaeodactylum tricornutum* (diatom)	5.81 mg/g	Gilbert-López et al., 2017
	Ethanol	100°C, 10.3 MPa	*Phaeodactylum tricornutum* (diatom)	26.1 mg/g	Derwenskus et al., 2019
UAE	Ethanol	25°C, 70 KHz, 30 min	*Phaeodactylum tricornutum* (diatom)	15.96 mg/g	Kim et al., 2012
	Acetone	8.5°C, 12.2 W, 10 min	*Cylindrotheca closterium* (diatom)	4.49 μg/g	Pasquet et al., 2011
	Coconut oil	450 W, 25 KHz, 15 min (bath)	*Phaeodactylum tricornutum* (diatom)	0.97 mg/mL	Papadaki et al., 2017
	Ethanol (80%, v/v)	230 W, 50 Hz, 30 min (bath)	*Padina tetrastromatica* (seaweed)	0.75 mg/g	Raguraman et al., 2018
MAE	Acetone	50 W, 5 min	*Cylindrotheca closterium* (diatom)	4.24 μg/mg	Pasquet et al., 2011
	Ethanol (50%)	300 W, 60°C, 10 min	*Laminaria japonica* (seaweed)	5.13 mg/100 g	Xiao et al., 2012
	Ethanol (50%)	300 W, 60°C, 10 min	*Undaria pinnatifida* (seaweed)	109.3 mg/100 g	Xiao et al., 2012
	Ethanol (50%)	300 W, 60°C, 10 min	*Sargassum fusiforme* (seaweed)	2.12 mg/100 g	Xiao et al., 2012
	Ethanol (50%)	300 W, 60°C, 5 min	*Undaria pinnatifida* (seaweed)	0.73 mg/g	Xiao et al., 2012
	Ethanol	850 W, 2455 MHz, 2 min	*Phaeodactylum tricornutum* (diatom)	4.59 mg/g	Gilbert-López et al., 2017
	Ethanol	700 W, 2450 MHz, 1 min	*Phaeodactylum tricornutum* (diatom)	58.07%	Zhang et al., 2018
EAE	Sodium buffer, Cellulase:pectinase	50°C, 80 min	*Laminaria japonica* (seaweed)	18.3 mg/100 g	Qin et al., 2013
	Sodium acetate buffer (0.1M), Viscozyme (100 fungal β-glucanase units/g)	50°C, 100 rpm, 12 h	*Fucus vesiculosus* (seaweed)	0.657 mg/g	Shannon and Abu-Ghannam, 2018

#### Supercritical Fluid Extraction (SFE)

Supercritical fluid extraction (SFE) utilizes carbon dioxide (CO_2_) at high pressure and constant temperature to extract bioactive components from feedstock (Kanda et al., 2014). Supercritical fluids had a better transport performance than liquid because of its low viscosity and high diffusivity (Kadam et al., 2013). Moreover, the dissolving power of supercritical fluid is dependent on its density, which is regulated by temperature and pressure (Kadam et al., 2013). The physical appearance of the final product is typically in oily and concentrated forms. This extraction method is deemed to be a sustainable processing method as it aligns with SDGs via the usage of environmentally benign solvents (Ramsey et al., 2009). The utilization of CO_2_ in its supercritical fluid state (SC-CO_2_) as an extraction solvent reduces the reliance on organic solvents and minimizes the generation of hazardous waste during processing. The low viscosity of SC-CO_2_ ensures a more efficient mass transfer for rapid penetration of solid matrices and extraction of compounds (Ramsey et al., 2009). More importantly, the solvating strength and polarity of SC-CO_2_ can be manipulated by controlling the density of SC-CO_2_, which can be regulated by the temperature and pressure (Kadam et al., 2013). The low critical temperature of CO_2_ (31°C) allows the extraction of carotenoid at a relatively lower temperature as compared to other traditional extraction methods involving high temperature (Ramsey et al., 2009).

A SFE study performed by Kanda et al. (2014) showed that the extraction of fucoxanthin from *Undaria pinnatifida* by SC-CO_2_ increased at least 16-fold in the presence of ethanol as an entrainer (3.23%). Similarly, the addition of entrainer (15% ethanol) was effective in the extraction of fucoxanthin from *Sargassum muticum* and the yield of fucoxanthin was improved marginally (Conde et al., 2014). Ethanol was commonly used as an entrainer in SC-CO_2_ extraction to increase the polarity of CO_2_; this effect is beneficial to the performance of fucoxanthin extraction. A recent work by Aslanbay Guler et al. (2020) showed that the yield of fucoxanthin extracted from *P. tricornutum* using SC-CO_2_ was 0.69 mg/g, which was comparable to the yield of fucoxanthin (0.57 mg/g) obtained by the conventional solvent extraction with methanol.

Yet, there are limitations in SFE of fucoxanthin because the optimal extraction conditions are dependent on the characteristics of algae species and fucoxanthin. For example, the vapor pressure of fucoxanthin is an important factor influencing the extraction efficiency; the high vapor pressure of fucoxanthin at a higher temperature enhances its diffusion from the solid matrices (Quitain et al., 2013). In addition, the algae biomass subjected to SFE must undergo an energy-intensive drying step because the water layers on wet biomass obstruct the penetration of SC-CO_2_ (Derwenskus et al., 2019). Therefore, the implementation of SFE for industrial applications may face some challenges. For example, the requirement of pressurized gas and the expensive equipment may impose greater operational and investment costs. Nonetheless, CO_2_ could be easily recycled by separating the gas stream during the process depressurization. The application of entrainer or co-solvents can improve the extraction efficiency of SFE but an additional step of solvent separation from the extract is required (Quitain et al., 2013). Moreover, polar impurities such as pigments may be co-extracted because the entrainer tends to enhance the polarity of SC-CO_2_.

#### Pressurised Liquid Extraction (PLE)

Pressurised liquid extraction (PLE) is an extraction technique utilizing high temperature (50–200°C) and pressure (3.5–20 MPa) to improve solubility and diffusion rate of biomolecules from complex crude extracts to the solvent phase (Kadam et al., 2013; Derwenskus et al., 2019). In PLE, the high-pressure condition increases the fluid density and maintains the solvent in the liquid (subcritical) state above their boiling point, while the high-temperature condition accelerates the penetration of solvents by lowering the viscosity and surface tension of solvents (Gilbert-López et al., 2017). The major advantages of PLE over the direct solvent extraction and Soxhlet extraction techniques include the rapid extraction process and the lower consumption of solvent. Ethanol was commonly used in the extraction of fucoxanthin via PLE. Although the high-temperature condition of PLE enhances the solubility and diffusivity characteristics of compounds, it was not favorable for the extraction of fucoxanthin from *Eisenia bicyclis* because of the yield of fucoxanthin was only about 0.42 mg/g (Shang et al., 2011). A similar observation was also reported by Gilbert-López et al. (2017), who discovered that the yield of fucoxanthin dropped by 25% when the operating temperature of PLE increased from 50 to 170°C. Fucoxanthin, which is a temperature-sensitive bioactive compound, can undergo oxidation process at high-temperature conditions and result in the poor yield of extraction. Therefore, it is envisaged that optimal operating temperature for the extraction of fucoxanthin is to be used to preserve the extracted fucoxanthin and subsequently its bioactivities. Furthermore, the safety of PLE operation must be considered because of the high pressure used. The operation period needs to be optimized for ensuring a sufficient contact time between fucoxanthin and solvents until the concentration gradient of fucoxanthin between solvent phase and plant matrix reached a balance. However, a prolonged period of PLE was not encouraged because fucoxanthin could undergo isomerization under the extreme physical conditions of PLE (Aslanbay Guler et al., 2020).

#### Ultrasound-Assisted Extraction (UAE)

Ultrasound-assisted extraction (UAE) has proven useful in overcoming the bottlenecks of conventional solvent extraction processes such as extraction duration and solvent consumption (Papadaki et al., 2017). This approach has been widely used for the extraction of various carotenoids and high-value bioactive compounds (e.g., lutein, astaxanthin, canthaxanthin, β-carotenes, docosahexaenoic acid, eicosapentaenoic acid) from complex feedstock (Cravotto et al., 2008; Dey and Rathod, 2013; Taghi Gharibzahedi et al., 2015; Goula et al., 2017; Chew et al., 2018; Sankaran et al., 2018). The ultrasound technology induces cavitation bubbles that collapse and produce heat energy along with ultrasonic waves (Chemat et al., 2017). These generated mechanical shear forces are responsible for disrupting the cell wall of algae, thereby releasing the target compounds into the solvent phase. The advantage of UAE lies in the disruptive-extractive forces that facilitate the extraction of target compounds from complex feedstock (e.g., algae with thick cell wall) in a single-step approach within a shorter period of extraction. Moreover, the mixing effect caused by acoustic streaming enhances the contact between solvents and target compounds. In conjunction with SDGs, ultrasound technology has been a potential extraction method for the green and sustainable processing of bioactive compounds from natural resources (Tiwari, 2015).

The UAE can be achieved with either an ultrasound bath or an ultrasound probe. It is recommended to adopt a probe instrument due to its effectiveness in cell disruption and energy efficiency. However, the drawback of the ultrasound probe is the overheating of the tip of ultrasound probe that could damage the heat-labile compounds. To overcome the overheating issue, the sample is usually chilled in an ice bath prior to the ultrasonic treatment. In general, UAE with an ultrasound probe gave a higher yield of fucoxanthin (15.96 mg/g), as compared to the ultrasound bath yielding only 0.75–0.97 mg/g of extracted fucoxanthin (Kim et al., 2012; Papadaki et al., 2017; Raguraman et al., 2018). To date, there is still insufficient literature reporting the application of UAE of fucoxanthin from diatom species.

#### Microwave-Assisted Extraction (MAE)

Microwave-assisted extraction (MAE) is a rapid and efficient extraction process for the recovery of bioactive compounds. The heating of the sample by microwave can be typically done in less than a minute, and the homogenous heating of the sample by microwave irradiation ensures no hot spots or limitations in heat transfer (Pasquet et al., 2011). Microwave irradiation induces heat energy through molecular interaction between solid and liquid (Chew et al., 2019). The heating effect generated from the incident electromagnetic waves promote the rapid dissolution of photosynthetic membranes by selective heating of the more polar part of cellulose (Banik et al., 2003). This heating effect is useful for releasing fucoxanthin from the fucoxanthin-chl *a*/*c*-protein complexes (Halim et al., 2012; Xia et al., 2013). Besides, the microwave facilitates an efficient release of intracellular bioactive compounds by improving the penetration of solvent into the matrix (Kadam et al., 2013). In aligning to the principles of green chemistry, microwave technology is favored for a green and clean process of extraction without the need for high-pressure conditions.

Ethanol has been commonly chosen as the solvent for MAE of fucoxanthin. This bio-based solvent favorably interacts with membrane-related lipid complexes. MAE of fucoxanthin in ethanol has been applied to macroalgae such as *L. japonica, Undaria pinnatifida*, and *Sargassum fusiforme* under the operating conditions of 300 W, 60°C, and 10 min. Among the tested macroalgae strains, the yield of fucoxanthin obtained from brown seaweed *S. fusiforme* was the least, which might be due to the rigidity of the cellular wall structure of algae. On the other hand, there are two separate studies demonstrating the rapid extraction of fucoxanthin from microalgae *P. tricornutum* via MAE in ethanol, which could be completed within 1–2 min (del Pilar Sánchez-Camargo et al., 2017; Zhang et al., 2018). The higher yield of fucoxanthin extracted from microalgae was attributed to the fact that cell wall of microalgae is less recalcitrant than that of macroalgae. To date, the scaling-up of MAE operation remains a challenge and the operating parameters such as temperature and duration of treatment must be optimized systematically. Preferably, the MAE of carotenoid should not exceed 60°C (Pasquet et al., 2011). The pulsed microwave processing or the continuous interval microwave processing with a short period of treatment time could circumvent the over-heating of sample, which effectively reduces the rate of fucoxanthin degradation during the MAE process.

#### Enzymatic-Assisted Extraction (EAE)

Enzymatic-assisted extraction (EAE) involves the use of hydrolytic enzymes such as pectinase and cellulase to hydrolyze algal cell walls. Algal cell walls mainly consist of cellulose (Domozych, 2001). The enzymatic treatment of algae is effective in hydrolyzing the cell wall to release the intracellular components into the extraction medium. Furthermore, EAE can be considered as a relatively low-cost technology if common food-grade enzymes including amylase, cellulose, pectinase, or β-galactosidase are used. In comparison to other alternative extraction techniques, EAE does not depend on energy-intensive equipment and it can be applied for large-scale extraction of algal bioactive compounds. However, EAE can be inadequate for large-scale applications due to the main drawbacks such as long enzymatic process, low selectivity, and poor yield. Apart from the duration of enzymatic treatment, the temperature condition of EAE must be optimized to maximize the extraction yield.

A recent work by Shannon and Abu−Ghannam (2018) demonstrated the applicability of EAE of fucoxanthin in brown seaweeds pre-treated at low temperature followed by drying and mechanical blending. A commercial enzyme cocktail, Viscozyme, was found to be effective not only in hydrolyzing the cellulose in the cell wall of seaweeds but also in reducing the viscosity of mixture. However, the efficiency of EAE was dependent on the physical texture and the target part (e.g., blade, stipe or holdfast) of seaweed. EAE could also be used as a pre-treatment step to improve the yield of fucoxanthin obtained from solvent extraction (dimethyl ether and ethanol); a 9.3% increase in fucoxanthin yield was obtained from *Undaria pinnatifida* biomass that was pre-treated by alginate lyase (Billakanti et al., 2013). EAE of fucoxanthin from microalgae was feasible, although the yield was lower as compared to that from the conventional extraction approaches.

## Future Perspectives: Potential and Limitation of Extraction Techniques

Conventional solid-liquid extraction has always been the dominant method used in the extraction of carotenoids from natural sources. Likewise, fucoxanthin is no exception, as evidenced by this review. Although solvents used in solid-liquid extraction could be recovered by evaporation or steam distillation processes for the economical consideration, it is desirable to minimize the usage of chemicals in the extraction process due to the inherent concerns over the toxicity of chemical solvents and the stricter regulations on food and environment. Moreover, increasing consumer awareness, as well as the urge for sustainable and responsible production, have put pressure on the search for cleaner and greener extraction processes. Considering the rising demand for bioactive compounds, the efforts in developing more efficient extraction technologies are underway. Irrespective of the extraction methods, it is crucial to ensure that the bioactivity of fucoxanthin is not compromised during the extraction.

The major challenge in fucoxanthin extraction is the potential degradation of fucoxanthin during the extraction process. Fortunately, it is apparent that the emerging extraction methods can be a good substitute to conventional extraction techniques, although none of them can be regarded as the prime method for the extraction of fucoxanthin from algal sources. In general, these alternative and emerging extraction methods have been proven effective in reducing the total volume of solvent consumed. Except for EAE, these extraction methods are energy-intensive, and their instrumentation and setup costs could be exorbitant to small and medium-sized enterprises. PLE and MAE methods are restricted by the high-temperature conditions used, and the optimization of extraction parameters are required for different algal species. EAE can mitigate the reliance on solvent, but the major obstacle to its application is the high cost of enzymes as compared to solvents and CO_2_. SFE and UAE methods are increasingly viewed as viable methods for the extraction of fucoxanthin. UAE is a versatile extraction method that can be integrated with other novel extraction methods such as SFE, MAE, and PLE. The integrative extraction method exploits the advantages of both discrete methods for a more satisfactory extraction performance.

SC-CO_2_ has gained popularity as a suitable solvent for the extraction of plant-based biomolecules because it is inexpensive, non-toxic, chemically inert, non-flammable, and readily available (Roh et al., 2008). Aside from that, an ongoing research effort has been devoted to the microalgae biorefinery by using green solvents like ionic liquids (ILs) and deep eutectic solvents (DESs). ILs have distinctive characteristics such as tunable properties, low melting point, low vapor pressure, and resistance toward high thermal stability. Surface-active ILs and tensioactive compounds have a high affinity to the hydrophobic carotenoid or pigments present in algal biomass (Vieira et al., 2018). They are miscible with water, making them suitable for extraction of fucoxanthin from wet algal biomass. The extractive capability of the anionic surfactant sodium dodecyl sulfate (SDS) was more superior than that of ethanol in the extraction of carotenoid (fucoxanthin included) from wet or dried biomass of *Sargassum muticum* (Vieira et al., 2018). It was postulated that SDS promoted cell disruption, facilitating the solubilization of hydrophobic compounds. However, the sustainable extraction of fucoxanthin using ILs still requires more in-depth studies as the concern for IL application is mainly on the hazardous raw materials involved in the synthesis of certain types of ILs. Alternative solvents such as using choline-based DESs have yet to be fully explored for fucoxanthin extraction, but the high viscosity of DESs may obstruct the mass transfer during extraction. Ideally, the recyclability of these alternative solvents should be adequately addressed to meet the sustainability criteria in the water-based extraction of fucoxanthin.

The extraction of fucoxanthin at a commercial scale is viable, as evidenced by a past study by Kanazawa et al. (2008). It was reported that the extraction of fucoxanthin from the waste part of brown macroalgae, *L. japonica* via a conventional extraction approach (i.e., ethanol along with silica gel column separation). The extracted fucoxanthin was estimated to be 149 g of fucoxanthin per ton of waste part of *L. japonica.* This biorefinery process is a good example of circular economy and it fulfills the SDGs by utilizing waste as a raw material of bioproduct. Liquefied dimethyl ether was also proposed as a suitable solvent for extraction for fucoxanthin due to its low boiling point for easy removal of solvent from the final product (Zarekarizi et al., 2019) as well as lower energy consumption. Moreover, liquefied dimethyl ether was regarded as a safe extraction solvent by food safety authorities from various countries (Zarekarizi et al., 2019). Aside from these, the scaling-up of extraction process must also consider the safety of operation at industrial level.

The biorefinery of microalgae for fucoxanthin extraction must be thoroughly considered in terms of techno-economic analysis (assessing economic feasibility) and life-cycle analysis (assessing potential environmental impacts). In particular, the de-watering step of microalgae biomass from culture (via energy-intensive centrifugation) prior to the extraction step is the major contributor to energy consumption and eventually increases total production cost. Therefore, direct extraction of fucoxanthin or other carotenoids from the wet microalgae biomass could overcome this concern. For example, fucoxanthin was extracted from wet *P. tricornutum* by subcritical fluid extraction, or known as PLE (Derwenskus et al., 2019; Aslanbay Guler et al., 2020). The optimal yield of fucoxanthin was attained by using methanol at solvent-to-solid ratio of 200:1, 200 MPa pressure and 35°C (Aslanbay Guler et al., 2020). The residual carbon-rich biomass obtained after the extraction could be prospectively used in other applications including livestock feed and fodder, making the extraction method a sustainable platform for biorefinery.

Furthermore, the isolation of fucoxanthin from algal sources could be accompanied by a step of purification to improve the purity of fucoxanthin. The conventional solvent extraction of fucoxanthin from brown algae could be non-selective and would co-extract chlorophylls during the extraction process. Fucoxanthin is often associated with the chlorophyll in the assembly of fucoxanthin-chlorophyll-protein complexes (Wang et al., 2005). This phenomenon renders a complex mixture for the downstream processing of fucoxanthin. Therefore, the ability of a solvent to specifically target fucoxanthin instead of other pigments from algal sources should be considered in these alternative and emerging extraction processes. Chromatographic separation techniques such as silica gel column chromatography, preparative thin-layer chromatography, high-performance liquid chromatography and liquid-liquid partition chromatography have been commonly used in the purification of fucoxanthin (Xiao et al., 2012). To improve the overall efficiency of downstream processing, the extraction techniques can be coupled with the separation techniques. The fucoxanthin-containing solvent fraction retrieved from the solvent extraction (e.g., ethanolic stream) could be directly subjected to the liquid-liquid separation systems like aqueous two-phase system (Gómez-Loredo et al., 2014, 2015) or high-speed countercurrent chromatography (Xiao et al., 2012). Prospectively, the simultaneous extraction and purification of fucoxanthin can be accomplished by integrating biphasic separation techniques with the emerging extraction techniques assisted by ultrasound, electricity, microwave, magnetic field and bubbles (Zhao et al., 2016; Sankaran et al., 2018; Chew et al., 2019; Khoo et al., 2019; Leong et al., 2019).

## Conclusion

With the inevitable increase in the human population, it is pertinent to ensure food security by seeking alternative and sustainable food sources. Microalgae stand out as a promising candidate as the next generation feedstock that contributes to 16 out of 17 SDGs. One of the most important microalgal biomolecules is fucoxanthin, which has powerful antioxidant properties due to its radical scavenging and singlet oxygen quenching activities. However, the apparent energy-intensive nature of emerging extraction methods can be prohibitive to the commercial production of fucoxanthin and counter-productive to the objectives of SDGs. From here, this review provides an updated understanding of existing and alternative green extraction technologies for fucoxanthin extraction. This enables stakeholders to make informed decisions which is an important milestone for biorefineries, propelling industries toward sustainable development.

The green extraction strategy is one of the many processing stages for sustainable supplies of bioactive ingredients. Other processing stages like extract separation and purification or upstream work (e.g., microalgae cultivation, biomass harvesting, transportation, and storage as well as a fair trade for workers) should also be aligned to SDGs. Further optimization of extraction technologies should focus on the minimization of energy consumption and the integration of different extraction methods to improve the efficiency of fucoxanthin extraction. In the long term, the gradual adoption and advocacy of sustainable strategies in microalgae biorefinery will ultimately close the loop for a circular bioeconomy.

## Author Contributions

SF conceptualized, wrote a section, and led the project. KK, CO, and PS contributed to the extraction processes section. NK contributed the section “Introduction.” FY completed the section “Conclusion.” All authors critically reviewed, edited, and approved the final manuscript.

## Conflict of Interest

The authors declare that the research was conducted in the absence of any commercial or financial relationships that could be construed as a potential conflict of interest.

## References

[B1] Abdel-Raouf N. Al-Homaidan A. Ibraheem I. (2012). Microalgae and wastewater treatment. *Saudi J. Biol. Sci.* 19 257–275. 10.1016/j.sjbs.2012.04.005 24936135 PMC4052567

[B2] Abu-Ghannam N. Shannon E. (2017). “Seaweed carotenoid, fucoxanthin, as functional food,” in *Microbial Functional Foods and Nutraceuticals*, eds Gupta V. K. Treichel H. Shapaval V. O. Antoniode Oliveira L. Tuohy M. G. (Chichester: John Wiley & Sons Ltd), 39–64. 10.1002/9781119048961.ch3

[B3] Agatonovic-Kustrin S. Morton D. (2013). Cosmeceuticals derived from bioactive substances found in marine algae. *Oceanography* 1:106.

[B4] Airanthi M. W. Hosokawa M. Miyashita K. (2011). Comparative antioxidant activity of edible japanese brown seaweeds. *J. Food Sci.* 76 C104–C111. 10.1111/j.1750-3841.2010.01915.x 21535637

[B5] Akinsemolu A. A. (2018). The role of microorganisms in achieving the sustainable development goals. *J. Cleaner Prod.* 182 139–155. 10.1016/j.jclepro.2018.02.081

[B6] Aslanbay Guler B. Deniz I. Demirel Z. Yesil-Celiktas O. Imamoglu E. (2020). A novel subcritical fucoxanthin extraction with a biorefinery approach. *Biochem. Eng. J.* 153:107403 10.1016/j.bej.2019.107403

[B7] Banerjee A. Sharma R. Chisti Y. Banerjee U. C. (2002). *Botryococcus braunii*: a renewable source of hydrocarbons and other chemicals. *Crit. Rev. Biotechnol.* 22 245–279. 10.1080/07388550290789513 12405558

[B8] Banik S. Bandyopadhyay S. Ganguly S. (2003). Bioeffects of microwave—-a brief review. *Bioresour. Technol.* 87 155–159. 10.1016/S0960-8524(02)00169-4 12765354

[B9] Batista A. P. Nunes M. C. Fradinho P. Gouveia L. Sousa I. Raymundo A. (2012). Novel foods with microalgal ingredients – Effect of gel setting conditions on the linear viscoelasticity of Spirulina and Haematococcus gels. *J. Food Eng.* 110 182–189. 10.1016/j.jfoodeng.2011.05.044

[B10] Bayro-Kaiser V. Nelson N. (2017). Microalgal hydrogen production: prospects of an essential technology for a clean and sustainable energy economy. *Photosynthesis Res.* 133 49–62. 10.1007/s11120-017-0350-6 28239761 PMC5500669

[B11] Beal C. M. Gerber L. N. Thongrod S. Phromkunthong W. Kiron V. Granados J. (2018). Marine microalgae commercial production improves sustainability of global fisheries and aquaculture. *Sci. Rep.* 8:15064. 10.1038/s41598-018-33504-w 30305674 PMC6180066

[B12] Beppu F. Niwano Y. Tsukui T. Hosokawa M. Miyashita K. (2009). Single and repeated oral dose toxicity study of fucoxanthin (FX), a marine carotenoid, in mice. *J. Toxicol. Sci.* 34 501–510. 10.2131/jts.34.501 19797858

[B13] Billakanti J. M. Catchpole O. J. Fenton T. A. Mitchell K. A. Mackenzie A. D. (2013). Enzyme-assisted extraction of fucoxanthin and lipids containing polyunsaturated fatty acids from *Undaria pinnatifida* using dimethyl ether and ethanol. *Process Biochem.* 48 1999–2008. 10.1016/j.procbio.2013.09.015

[B14] Biloria N. Thakkar Y. (2020). Integrating algae building technology in the built environment: a cost and benefit perspective. *Front. Architect. Res.* 9 370–384. 10.1016/j.foar.2019.12.004

[B15] Bjornland T. Liaeen-Jensen S. (1989). “Distribution patterns of carotenoids in relation to chromophyte phylogeny and systematics,” in *The Chromophyte Algae: Problems and Perspectives*, eds Green J. C. Leadbeater B. S. C. Diver W. L. (Oxford: Clarendon Press), 37–61.

[B16] Blackburn S. Bolch C. Brown M. Leroi J.-M. Volkman J. (1997). *The CSIRO Collection of Living Microalgae: The Importance of Culture Collections and Microalgal Biodiversity to Aquaculture and Biotehcnology.* (Plouzane: IFREMER).

[B17] Blackburn S. Frampton D. Jameson I. Brown M. Mansour M. Negri A. (2005). “The CSIRO collection of living microalgae: an australian perspective on microalgal biodiversity and applications,” in *Algal Culture Collections and the Environment*, eds Kasai F. Kaya K. Watanabe M. M. (Hadano: Tokai University Press), 29–63.

[B18] Blanco A. C. Nadaoka K. Yamamoto T. (2008). Planktonic and benthic microalgal community composition as indicators of terrestrial influence on a fringing reef in Ishigaki Island, Southwest Japan. *Mar. Environ. Res.* 66 520–535. 10.1016/j.marenvres.2008.08.005 18849068

[B19] Chemat F. Rombaut N. Sicaire A.-G. Meullemiestre A. Fabiano-Tixier A.-S. Abert-Vian M. (2017). Ultrasound assisted extraction of food and natural products. Mechanisms, techniques, combinations, protocols and applications. A review. *Ultrason. Sonochem.* 34 540–560. 10.1016/j.ultsonch.2016.06.035 27773280

[B20] Chew K. W. Chia S. R. Lee S. Y. Zhu L. Show P. L. (2019). Enhanced microalgal protein extraction and purification using sustainable microwave-assisted multiphase partitioning technique. *Chem. Eng. J.* 367 1–8. 10.1016/j.cej.2019.02.131

[B21] Chew K. W. Show P. L. Yap Y. J. Juan J. C. Phang S. M. Ling T. C. (2018). Sonication and grinding pre-treatments on *Gelidium amansii* seaweed for the extraction and characterization of agarose. *Front. Environ. Sci. Eng.* 12:2 10.1007/s11783-018-1040-0

[B22] Conde E. Moure A. Domínguez H. (2014). Supercritical CO2 extraction of fatty acids, phenolics and fucoxanthin from freeze-dried *Sargassum muticum*. *J. Appl. Phycol.* 27 957–964. 10.1007/s10811-014-0389-0

[B23] Couteau C. Coiffard L. (2016). “Seaweed application in cosmetics,” in *Seaweed in Health and Disease Prevention*, eds Fleurence J. Levine I. (Amsterdam: Elsevier), 423–441. 10.1016/B978-0-12-802772-1.00014-2

[B24] Cravotto G. Boffa L. Mantegna S. Perego P. Avogadro M. Cintas P. (2008). Improved extraction of vegetable oils under high-intensity ultrasound and/or microwaves. *Ultrason. Sonochem.* 15 898–902. 10.1016/j.ultsonch.2007.10.009 18093864

[B25] Dahoumane S. A. Mechouet M. Alvarez F. J. Agathos S. N. Jeffryes C. (2016). Microalgae: an outstanding tool in nanotechnology. *Bionatura* 1 196–201. 10.21931/RB/2016.01.04.7

[B26] del Pilar Sánchez-Camargo A. Pleite N. Herrero M. Cifuentes A. Ibáñez E. Gilbert-López B. (2017). New approaches for the selective extraction of bioactive compounds employing bio-based solvents and pressurized green processes. *J. Supercrit. Fluids* 128 112–120. 10.1016/j.supflu.2017.05.016

[B27] Derun Y. (2009). “Training of trainers programme: strengthening capacity of small holder ASEAN aquaculture farmers for competitive and sustainable aquaculture,” *Proceedings of a 3–7 August 2009 Conference* (Bangkok: NACA Secretariat), Available online at: http://library.enaca.org/inland/reports/training-oftrainers-report.pdf

[B28] Derwenskus F. Metz F. Gille A. Schmid-Staiger U. Briviba K. Schließmann U. (2019). Pressurized extraction of unsaturated fatty acids and carotenoids from wet *Chlorella vulgaris* and *Phaeodactylum tricornutum* biomass using subcritical liquids. *GCB Bioenergy* 11 335–344. 10.1111/gcbb.12563

[B29] Dey S. Rathod V. K. (2013). Ultrasound assisted extraction of β-carotene from *Spirulina platensis*. *Ultrason. Sonochem.* 20 271–276. 10.1016/j.ultsonch.2012.05.010 22705076

[B30] Domozych D. S. (2001). “Algal cell walls,” in *eLS*, ed. P. Gregg (Hoboken, NJ: Wiley). 10.1038/npg.els.0000315

[B31] Ebrahimi Nigjeh S. Yusoff F. M. Mohamed Alitheen N. B. Rasoli M. Keong Y. S. Omar A. R. B. (2013). Cytotoxic effect of ethanol extract of microalga, Chaetoceros calcitrans, and its mechanisms in inducing apoptosis in human breast cancer cell line. *Biomed Res. Int.* 2013:783690. 10.1155/2013/783690 23509778 PMC3591159

[B32] Elrayies G. M. (2018). Microalgae: prospects for greener future buildings. *Renew. Sustain. Energy Rev.* 81 1175–1191. 10.1016/j.rser.2017.08.032

[B33] Foo S. C. Yusoff F. M. Imam M. U. Foo J. B. Ismail N. Azmi N. H. (2019). Increased fucoxanthin in *Chaetoceros calcitrans* extract exacerbates apoptosis in liver cancer cells via multiple targeted cellular pathways. *Biotechnol. Rep.* 21:e00296. 10.1016/j.btre.2018.e00296 30581767 PMC6296166

[B34] Foo S. C. Yusoff F. M. Ismail M. Basri M. Chan K. W. Khong N. M. H. (2015a). Production of fucoxanthin-rich fraction (FxRF) from a diatom, Chaetoceros calcitrans (Paulsen) Takano 1968. *Algal Res.* 12 26–32. 10.1016/j.algal.2015.08.004

[B35] Foo S. C. Yusoff F. M. Ismail M. Basri M. Khong N. M. H. Chan K. W. (2015b). Efficient solvent extraction of antioxidant-rich extract from a tropical diatom, *Chaetoceros calcitrans* (Paulsen) Takano 1968. *Asian Pac. J. Trop. Biomed.* 5 834–840. 10.1016/j.apjtb.2015.06.003

[B36] Foo S. C. Yusoff F. M. Ismail M. Basri M. Yau S. K. Khong N. M. (2017). Antioxidant capacities of fucoxanthin-producing algae as influenced by their carotenoid and phenolic contents. *J. Biotechnol.* 241 175–183. 10.1016/j.jbiotec.2016.11.026 27914891

[B37] Gaignard C. Laroche C. Pierre G. Dubessay P. Delattre C. Gardarin C. (2019). Screening of marine microalgae: investigation of new exopolysaccharide producers. *Algal Res.* 44 101711 10.1016/j.algal.2019.101711

[B38] García J. L. De Vicente M. Galán B. (2017). Microalgae, old sustainable food and fashion nutraceuticals. *Microb. Biotechnol.* 10 1017–1024. 10.1111/1751-7915.12800 28809450 PMC5609256

[B39] Getachew A. T. Saravana P. S. Cho Y. J. Woo H. C. Chun B. S. (2018). Concurrent extraction of oil from roasted coffee (*Coffea arabica*) and fucoxanthin from brown seaweed (*Saccharina japonica*) using supercritical carbon dioxide. *J. CO2 Util.* 25 137–146. 10.1016/j.jcou.2018.03.018

[B40] Gilbert-López B. Barranco A. Herrero M. Cifuentes A. Ibáñez E. (2017). Development of new green processes for the recovery of bioactives from *Phaeodactylum tricornutum*. *Food Res. Int.* 99 1056–1065. 10.1016/j.foodres.2016.04.022 28865617

[B41] Goiris K. Muylaert K. Fraeye I. Foubert I. De Brabanter J. De Cooman L. (2012). Antioxidant potential of microalgae in relation to their phenolic and carotenoid content. *J. Appl. Phycol.* 24 1477–1486. 10.1007/s10811-012-9804-6

[B42] Gómez-Loredo A. Benavides J. Rito-Palomares M. (2014). Partition behavior of fucoxanthin in ethanol-potassium phosphate two-phase systems. *J. Chem. Technol. Biotechnol.* 89 1637–1645. 10.1002/jctb.4514

[B43] Gómez-Loredo A. González-Valdez J. Rito-Palomares M. (2015). Insights on the downstream purification of fucoxanthin, a microalgal carotenoid, from an aqueous two-phase system stream exploiting ultrafiltration. *J. Appl. Phycol.* 27 1517–1523. 10.1007/s10811-014-0443-y

[B44] Goto M. Kanda H. Machmudah S. (2015). Extraction of carotenoids and lipids from algae by supercritical CO2 and subcritical dimethyl ether. *J. Supercrit. Fluids* 96 245–251. 10.1016/j.supflu.2014.10.003

[B45] Goula A. M. Ververi M. Adamopoulou A. Kaderides K. (2017). Green ultrasound-assisted extraction of carotenoids from pomegranate wastes using vegetable oils. *Ultrason. Sonochem.* 34 821–830. 10.1016/j.ultsonch.2016.07.022 27773309

[B46] Grant B. Waller R. F. Clementson L. A. Wetherbee R. (2013). Psammamonas australis gen. et sp. nov.(Raphidophyceae), a new dimorphic, sand-dwelling alga. *Phycologia* 52 57–64. 10.2216/12-070.1

[B47] Griffiths M. J. Harrison S. T. L. (2009). Lipid productivity as a key characteristic for choosing algal species for biodiesel production. *J. Appl. Phycol.* 21 493–507. 10.1007/s10811-008-9392-7

[B48] Halim R. Danquah M. K. Webley P. A. (2012). Extraction of oil from microalgae for biodiesel production: a review. *Biotechnol. Adv.* 30 709–732. 10.1016/j.biotechadv.2012.01.001 22266377

[B49] Hashimoto T. Ozaki Y. Taminato M. Das S. K. Mizuno M. Yoshimura K. (2009). The distribution and accumulation of fucoxanthin and its metabolites after oral administration in mice. *Br. J. Nutr.* 102 242–248. 10.1017/S0007114508199007 19173766

[B50] Hemaiswarya S. Raja R. Kumar R. R. Ganesan V. Anbazhagan C. (2011). Microalgae: a sustainable feed source for aquaculture. *World J. Microbiol. Biotechnol.* 27 1737–1746. 10.1007/s11274-010-0632-z

[B51] Heo S. J. Jeon Y. J. (2009). Protective effect of fucoxanthin isolated from *Sargassum siliquastrum* on UV-B induced cell damage. *J. Photochem. Photobiol. B Biol.* 95 101–107. 10.1016/j.jphotobiol.2008.11.011 19264501

[B52] Heo S. J. Yoon W. J. Kim K. N. Oh C. Choi Y. U. Yoon K. T. (2012). Anti-inflammatory effect of fucoxanthin derivatives isolated from *Sargassum siliquastrum* in lipopolysaccharide-stimulated RAW 264.7 macrophage. *Food Chem. Toxicol.* 50 3336–3342. 10.1016/j.fct.2012.06.025 22735499

[B53] Holdt S. L. Kraan S. (2011). Bioactive compounds in seaweed: functional food applications and legislation. *J. Appl. Phycol.* 23 543–597. 10.1007/s10811-010-9632-5

[B54] Jaswir I. Noviendri D. Salleh H. M. Taher M. Miyashita K. Ramli N. (2013). Analysis of fucoxanthin content and purification of all-trans-fucoxanthin from *Turbinaria turbinata* and *Sargassum plagyophyllum* by SiO2 open column chromatography and reversed phase-HPLC. *J. Liquid Chromatogr. Relat. Technol.* 36 1340–1354. 10.1080/10826076.2012.691435

[B55] Jelić S. Jovanović T. (2013). “Education in transition,” in *Terms Of The Development Of Agriculture And Rural Development.* ed. Bogdanov N. (Belgrade: University of Belgrada).

[B56] Kadam S. U. Tiwari B. K. O’donnell C. P. (2013). Application of novel extraction technologies for bioactives from marine algae. *J. Agric. Food Chem.* 61 4667–4675. 10.1021/jf400819p 23634989

[B57] Kanazawa K. Ozaki Y. Hashimoto T. Das S. K. Matsushita S. Hirano M. (2008). Commercial-scale preparation of biofunctional fucoxanthin from waste parts of brown sea algae *Laminalia japonica*. *Food Sci. Technol. Res.* 14 573–582. 10.3136/fstr.14.573

[B58] Kanda H. Kamo Y. Machmudah S. Goto M. (2014). Extraction of fucoxanthin from raw macroalgae excluding drying and cell wall disruption by liquefied dimethyl ether. *Mar. Drugs* 12 2383–2396. 10.3390/md12052383 24796299 PMC4052295

[B59] Kashyap M. Samadhiya K. Ghosh A. Anand V. Shirage P. M. Bala K. (2019). Screening of microalgae for biosynthesis and optimization of Ag/AgCl nano hybrids having antibacterial effect. *RSC Adv.* 9 25583–25591. 10.1039/C9RA04451E PMC9070394 35530087

[B60] Kavitha S. Gunasekaran M. (2020). Microalgae based biorefinery promoting circular bioeconomy-Techno economic and life-cycle analysis. *Bioresour. Technol.* 302:122822. 10.1016/j.biortech.2020.122822 32007307

[B61] Kawee-Ai A. Kim A. T. Kim S. M. (2019). Inhibitory activities of microalgal fucoxanthin against α-amylase, α-glucosidase, and glucose oxidase in 3T3-L1 cells linked to type 2 diabetes. *J. Oceanol. Limnol.* 37 928–937. 10.1007/s00343-019-8098-9

[B62] Khoo K. S. Chew K. W. Ooi C. W. Ong H. C. Ling T. C. Show P. L. (2019). Extraction of natural astaxanthin from *Haematococcus pluvialis* using liquid biphasic flotation system. *Bioresour. Technol.* 290:121794. 10.1016/j.biortech.2019.121794 31319214

[B63] Kim S.-K. (2011). *Marine Cosmeceuticals: Trends and Prospects.* Boca Raton, FL: CRC Press.

[B64] Kim S. K. (2012). *Handbook of Marine Macroalgae.* West Sussex: John Wiley & Sons Ltd.

[B65] Kim S. M. Jung Y.-J. Kwon O.-N. Cha K. H. Um B.-H. Chung D. (2012). A potential commercial source of fucoxanthin extracted from the microalga *Phaeodactylum tricornutum*. *Appl. Biochem. Biotechnol.* 166 1843–1855. 10.1007/s12010-012-9602-2 22371063

[B66] Kumar K. S. Dahms H.-U. Won E.-J. Lee J.-S. Shin K.-H. (2015). Microalgae–a promising tool for heavy metal remediation. *Ecotoxicol. Environ. Saf.* 113 329–352. 10.1016/j.ecoenv.2014.12.019 25528489

[B67] Kusin O.-S. C. Horan N. (2015). Energy and revenue creation from the anaerobic digestion of *Chlorella vulgaris* cultivated in liquor from chemically treated sewage sludge. *Int. J. Tech. Res. Appl.* 32 49–55.

[B68] Kwon K.-C. Lamb A. Fox D. Jegathese S. J. P. (2019). An evaluation of microalgae as a recombinant protein oral delivery platform for fish using green fluorescent protein (GFP). *Fish Shellfish Immunol.* 87 414–420. 10.1016/j.fsi.2019.01.038 30703550

[B69] Lababpour A. (2016). Potentials of the microalgae inoculant in restoration of biological soil crusts to combat desertification. *Int. J. Environ. Sci. Technol.* 13 2521–2532. 10.1007/s13762-016-1074-4

[B70] Leong H. Y. Ooi C. W. Law C. L. Julkifle A. L. Katsuda T. Show P. L. (2019). Integration process for betacyanins extraction from peel and flesh of Hylocereus polyrhizus using liquid biphasic electric flotation system and antioxidant activity evaluation. *Sep. Purif. Technol.* 209 193–201. 10.1016/j.seppur.2018.07.040

[B71] Lindblad P. Fuente D. Borbe F. Cicchi B. Conejero J. A. Couto N. (2019). CyanoFactory, a European consortium to develop technologies needed to advance cyanobacteria as chassis for production of chemicals and fuels. *Algal Res.* 41:101510 10.1016/j.algal.2019.101510

[B72] Liu C. L. Chiu Y. T. Hu M. L. (2011). Fucoxanthin enhances HO-1 and NQO1 expression in murine hepatic BNL CL. 2 cells through activation of the Nrf2/ARE system partially by its pro-oxidant activity. *J. Agric. Food Chem.* 59 11344–11351. 10.1021/jf2029785 21919437

[B73] Mace G. M. Norris K. Fitter A. H. (2012). Biodiversity and ecosystem services: a multilayered relationship. *Trends Ecol. Evol.* 27 19–26. 10.1016/j.tree.2011.08.006 21943703

[B74] Maeda H. Hosokawa M. Sashima T. Funayama K. Miyashita K. (2007). Effect of medium-chain triacylglycerols on anti-obesity effect of fucoxanthin. *J. Oleo Sci.* 56 615–621. 10.5650/jos.56.615 17992001

[B75] Matondo F. K. Takaisi K. Nkuadiolandu A. B. Kazadi Lukusa A. Aloni M. N. (2016). Spirulina supplements improved the nutritional status of undernourished children quickly and significantly: experience from kisantu, the Democratic Republic of the Congo. *Int. J. Pediatr.* 2016:1296414. 10.1155/2016/1296414 27777589 PMC5061973

[B76] McHugh D. J. (2003). *A Guide to the Seaweed Industry.* Rome: FAO.

[B77] Miyashita K. Nishikawa S. Hosokawa M. (2012). Therapeutic effect of fucoxanthin on metabolic syndrome and type 2 diabetes. *Nutr. Ther. Interv. Diabetes Metab. Syndr.* 3 367–379. 10.1016/B978-0-12-385083-6.00029-2

[B78] Moejes F. W. Moejes K. B. (2017). Algae for Africa: microalgae as a source of food, feed and fuel in Kenya. *Afr. J. Biotechnol.* 16 288–301. 10.5897/AJB2016.15721

[B79] Mohseniazar M. Barin M. Zarredar H. Alizadeh S. Shanehbandi D. (2011). Potential of microalgae and lactobacilli in biosynthesis of silver nanoparticles. *Bioimpacts* 1 149–152. 23678420 10.5681/bi.2011.020 PMC3648959

[B80] Moriarty P. Honnery D. (2019). New energy technologies: microalgae, photolysis and airborne wind turbines. *Sci* 1:43 10.3390/sci1020043

[B81] Muradian K. Vaiserman A. Min K.-J. Fraifeld V. (2015). Fucoxanthin and lipid metabolism: a minireview. *Nutr. Metab. Cardiovasc. Dis.* 25 891–897. 10.1016/j.numecd.2015.05.010 26141943

[B82] Neumann U. Derwenskus F. Gille A. Louis S. Schmid-Staiger U. Briviba K. (2018). Bioavailability and Safety of Nutrients from the Microalgae *Chlorella vulgaris*, *Nannochloropsis oceanica* and *Phaeodactylum tricornutum* in C57BL/6 Mice. *Nutrients* 10:965. 10.3390/nu10080965 30049974 PMC6116023

[B83] Nomura T. Kikuchi M. Kubodera A. Kawakami Y. (1997). Proton-donative antioxidant activity of fucoxanthin with 1,1-diphenyl-2-picrylhydrazyl (DPPH). *Biochem. Mol. Biol. Int.* 42 361–370. 10.1080/15216549700202761 9238535

[B84] Norton T. A. Melkonian M. Andersen R. A. (1996). Algal biodiversity^∗^. *Phycologia* 35 308–326. 10.2216/i0031-8884-35-4-308.1

[B85] Noviendri D. Jaswir I. Salleh H. M. Taher M. Miyashita K. Ramli N. (2011). Fucoxanthin extraction and fatty acid analysis of *Sargassum binderi* and *Sargassum duplicatum*. *J. Med. Plants Res.* 5 2405–2412.

[B86] Papadaki S. Kyriakopoulou K. Krokida M. (2017). Recovery and encapsualtion of bioactive extracts from *Haematococcus pluvialis* and *Phaedodactylum tricornutum* for food applications. *IOSR J. Environ. Sci. Toxicol. Food Technol.* 10 53–58.

[B87] Pasquet V. Chérouvrier J.-R. Farhat F. Thiéry V. Piot J.-M. Bérard J.-B. (2011). Study on the microalgal pigments extraction process: performance of microwave assisted extraction. *Process Biochem.* 46 59–67. 10.1016/j.procbio.2010.07.009

[B88] Peng J. Yuan J.-P. Wu C.-F. Wang J.-H. (2011). Fucoxanthin, a marine carotenoid present in brown seaweeds and diatoms: metabolism and bioactivities relevant to human health. *Mar. Drugs* 9 1806–1828. 10.3390/md9101806 22072997 PMC3210606

[B89] Prabhasankar P. Ganesan P. Bhaskar N. Hirose A. Stephen N. Gowda L. R. (2009). Edible Japanese seaweed, wakame (*Undaria pinnatifida*) as an ingredient in pasta: chemical, functional and structural evaluation. *Food Chem.* 115 501–508. 10.1016/j.foodchem.2008.12.047

[B90] Pulz O. Gross W. (2004). Valuable products from biotechnology of microalgae. *Appl. Microbiol. Biotechnol.* 65 635–648. 10.1007/s00253-004-1647-x 15300417

[B91] Qin Y. Meng L.-Y. Wang F.-W. (2013). Extraction and antioxidant activity of fucoxanthin from *Laminaria japonica*. *Food Sci.* 34 279–283.

[B92] Qiu F. (2014). *Algae Architecture.* Delft: Delft University of Technology.

[B93] Quitain A. T. Kai T. Sasaki M. Goto M. (2013). Supercritical carbon dioxide extraction of fucoxanthin from *Undaria pinnatifida*. *J. Agric. Food Chem.* 61 5792–5797. 10.1021/jf400740p 23742680

[B94] Raguraman V. Mubarakali D. Narendrakumar G. Thirugnanasambandam R. Kirubagaran R. Thajuddin N. (2018). Unraveling rapid extraction of fucoxanthin from *Padina tetrastromatica*: purification, characterization and biomedical application. *Process Biochem.* 73 211–219. 10.1016/j.procbio.2018.08.006

[B95] Ramsey E. Sun Q. Zhang Z. Zhang C. Gou W. (2009). Mini-review: green sustainable processes using supercritical fluid carbon dioxide. *J. Environ. Sci.* 21 720–726. 10.1016/S1001-0742(08)62330-X 19803072

[B96] Richmond A. (2017). “Microalgae of economic potential,” in *Handbook of Microalgal Mass Culture*, ed. A. Richmond (Boca Raton, FL: CRC Press), 199–244.

[B97] Roesijadi G. Jones S. B. Snowden-Swan L. J. Zhu Y. (2010). *Macroalgae as a Biomass Feedstock: A Preliminary Analysis.* Richland, WA: Pacific Northwest National Lab.(PNNL). 10.2172/1006310

[B98] Roh M.-K. Uddin M. S. Chun B.-S. (2008). Extraction of fucoxanthin and polyphenol from *Undaria pinnatifida* using supercritical carbon dioxide with co-solvent. *Biotechnol. Bioprocess Eng.* 13 724–729. 10.1007/s12257-008-0104-6

[B99] Sachindra N. M. Sato E. Maeda H. Hosokawa M. Niwano Y. Kohno M. (2007). Radical scavenging and singlet oxygen quenching activity of marine carotenoid fucoxanthin and its metabolites. *J. Agric. Food Chem.* 55 8516–8522. 10.1021/jf071848a 17894451

[B100] Sachs J. D. (2012). From millennium development goals to sustainable development goals. *Lancet* 379 2206–2211. 10.1016/S0140-6736(12)60685-0 22682467

[B101] Sankaran R. Manickam S. Yap Y. J. Ling T. C. Chang J.-S. Show P. L. (2018). Extraction of proteins from microalgae using integrated method of sugaring-out assisted liquid biphasic flotation (LBF) and ultrasound. *Ultrason. Sonochem.* 48 231–239. 10.1016/j.ultsonch.2018.06.002 30080546

[B102] Serrà A. Artal R. García-Amorós J. Sepúlveda B. Gómez E. Nogués J. (2020). Hybrid Ni@ ZnO@ ZnS-Microalgae for Circular economy: a smart route to the efficient integration of solar photocatalytic water decontamination and bioethanol production. *Adv. Sci.* 7:1902447. 10.1002/advs.201902447 32042564 PMC7001628

[B103] Shahidi F. Alasalvar C. (2010). “Marine Oils and other Marine Nutraceuticals,” in *Handbook of Seafood Quality, Safety and Health Applications*, eds Alasalvar C. Shahidi F. Miyashita K. Wanasundara U. (Hoboken, NJ: Wiley), 444–454. 10.1002/9781444325546.ch36

[B104] Shang Y. F. Kim S. M. Lee W. J. Um B.-H. (2011). Pressurized liquid method for fucoxanthin extraction from *Eisenia bicyclis* (Kjellman) Setchell. *J. Biosci. Bioeng.* 111 237–241. 10.1016/j.jbiosc.2010.10.008 21081286

[B105] Shannon E. Abu-Ghannam N. (2017). Optimisation of fucoxanthin extraction from Irish seaweeds by response surface methodology. *J. Appl. Phycol.* 29 1027–1036. 10.1007/s10811-016-0983-4

[B106] Shannon E. Abu-Ghannam N. (2018). Enzymatic extraction of fucoxanthin from brown seaweeds. *Int. J. Food Sci. Technol.* 53 2195–2204. 10.1111/ijfs.13808

[B107] Shen C.-T. Chen P.-Y. Wu J.-J. Lee T.-M. Hsu S.-L. Chang C.-M. J. (2011). Purification of algal anti-tyrosinase zeaxanthin from Nannochloropsis oculata using supercritical anti-solvent precipitation. *J. Supercrit. Fluids* 55 955–962. 10.1016/j.supflu.2010.10.003

[B108] Simas-Rodrigues C. Villela H. D. Martins A. P. Marques L. G. Colepicolo P. Tonon A. P. (2015). Microalgae for economic applications: advantages and perspectives for bioethanol. *J. Exp. Bot.* 66 4097–4108. 10.1093/jxb/erv130 25873683

[B109] Singh J. Tiwari O. N. Dhar D. W. (2019). Overview of carbon capture technology: microalgal biorefinery concept and state-of-the-art. *Front. Mar. Sci.* 6:29 10.3389/fmars.2019.00029

[B110] Sivagnanam S. P. Yin S. Choi J. H. Park Y. B. Woo H. C. Chun B. S. (2015). Biological properties of fucoxanthin in oil recovered from two brown seaweeds using supercritical CO2 extraction. *Mar. Drugs* 13 3422–3442. 10.3390/md13063422 26035021 PMC4483637

[B111] Stahl W. Sies H. (2012). ß-Carotene and other carotenoids in protection from sunlight. *Am. J. Clin. Nutr.* 96 1179S–1184S. 10.3945/ajcn.112.034819 23053552

[B112] Stauber J. L. Jeffrey S. W. (1988). Photosynthetic pigments in fifty-one species of marine diatoms. *J. Phycol.* 24 158–172. 10.1111/j.1529-8817.1988.tb04230.x

[B113] Taghi Gharibzahedi S. M. Razavi S. H. Mousavi M. (2015). Optimal development of a new stable nutraceutical nanoemulsion based on the inclusion complex of 2-hydroxypropyl-β-cyclodextrin with canthaxanthin accumulated by *Dietzia natronolimnaea* HS-1 using ultrasound-assisted emulsification. *J. Dispers. Sci. Technol.* 36 614–625. 10.1080/01932691.2014.921188

[B114] Takashima M. Shichiri M. Hagihara Y. Yoshida Y. Niki E. (2012). Capacity of fucoxanthin for scavenging peroxyl radicals and inhibition of lipid peroxidation in model systems. *Free Radic. Res.* 46 1406–1412. 10.3109/10715762.2012.721542 22900899

[B115] Talebi A. F. Tabatabaei M. Aghbashlo M. Movahed S. Hajjari M. Golabchi M. (2020). “Algae-powered buildings: a strategy to mitigate climate change and move toward circular economy,” in *Smart Village Technology*, eds Patnaik S. et al. (Cham: Springer), 353–365. 10.1007/978-3-030-37794-6_18

[B116] Tan C. P. Hou Y. H. (2014). First evidence for the anti-inflammatory activity of fucoxanthin in high-fat-diet-induced obesity in mice and the antioxidant functions in PC12 cells. *Inflammation* 37 443–450. 10.1007/s10753-013-9757-1 24146106

[B117] Tiwari B. K. (2015). Ultrasound: a clean, green extraction technology. *Trends Analyt. Chem.* 71 100–109. 10.1016/j.trac.2015.04.013

[B118] Toledo Á. Burlingame B. (2006). Biodiversity and nutrition: a common path toward global food security and sustainable development. *J. Food Composit. Anal.* 19 477–483. 10.1016/j.jfca.2006.05.001

[B119] Tredici M. R. Rodolfi L. Biondi N. Bassi N. Sampietro G. (2016). Techno-economic analysis of microalgal biomass production in a 1-ha Green Wall Panel (GWP^®^) plant. *Algal Res.* 19 253–263. 10.1016/j.algal.2016.09.005

[B120] Urikura I. Sugawara T. Hirata T. (2011). Protective effect of fucoxanthin against UVB-induced skin photoaging in hairless mice. *Biosci. Biotechnol. Biochem.* 75 757–760. 10.1271/bbb.110040 21512228

[B121] Van Harmelen T. Oonk H. (2006). *Microalgae Biofixation Processes: Applications and Potential Contributions to Greenhouse Gas Mitigation Options.* Apeldoorn: TNO Built Environmental Geosciences.

[B122] Vieira F. A. Guilherme R. J. R. Neves M. C. Rego A. Abreu M. H. Coutinho J. A. P. (2018). Recovery of carotenoids from brown seaweeds using aqueous solutions of surface-active ionic liquids and anionic surfactants. *Sep. Purif. Technol.* 196 300–308. 10.1016/j.seppur.2017.05.006

[B123] Wang W.-J. Wang G.-C. Zhang M. Tseng C. K. (2005). Isolation of fucoxanthin from the rhizoid of *Laminaria japonica* Aresch. *J. Integr. Plant Biol.* 47 1009–1015. 10.1111/j.1744-7909.2005.00054.x

[B124] Withers N. W. Fiksdahl A. Tuttle R. C. Liaaen-Jensen S. (1981). Carotenoids of the Chrysophyceae. *Comp. Biochem. Physiol. B* 68 345–349. 10.1016/0305-0491(81)90110-3

[B125] Wright S. W. Jeffrey S. W. (1987). Fucoxanthin pigment markers of marine phytoplankton analysed by HPLC and HPTLC. *Mar. Ecol. Prog. Ser.* 38 259–266. 10.3354/meps038259

[B126] Wu H. Li T. Wang G. Dai S. He H. Xiang W. (2015). A comparative analysis of fatty acid composition and fucoxanthin content in six *Phaeodactylum tricornutum* strains from different origins. *Chin. J. Oceanol. Limnol.* 38 391–398. 10.1007/s00343-015-4325-1

[B127] Xia S. Wang K. Wan L. Li A. Hu Q. Zhang C. (2013). Production, characterization, and antioxidant activity of fucoxanthin from the marine diatom *Odontella aurita*. *Mar. Drugs* 11 2667–2681. 10.3390/md11072667 23880936 PMC3736445

[B128] Xiao X. Si X. Yuan Z. Xu X. Li G. (2012). Isolation of fucoxanthin from edible brown algae by microwave-assisted extraction coupled with high-speed countercurrent chromatography. *J. Sep. Sci.* 35 2313–2317. 10.1002/jssc.201200231 22807438

[B129] Yan N. Fan C. Chen Y. Hu Z. (2016). The potential for microalgae as bioreactors to produce pharmaceuticals. *Int. J. Mol. Sci.* 17:962. 10.3390/ijms17060962 27322258 PMC4926494

[B130] Zarekarizi A. Hoffmann L. Burritt D. (2019). Approaches for the sustainable production of fucoxanthin, a xanthophyll with potential health benefits. *J. Appl. Phycol.* 31 281–299. 10.1007/s10811-018-1558-3

[B131] Zhang W. Wang F. Gao B. Huang L. Zhang C. (2018). An integrated biorefinery process: stepwise extraction of fucoxanthin, eicosapentaenoic acid and chrysolaminarin from the same *Phaeodactylum tricornutum* biomass. *Algal Res.* 32 193–200. 10.1016/j.algal.2018.04.002

[B132] Zhao X. Fu L. Liu D. Zhu H. Wang X. Bi Y. (2016). Magnetic-field-assisted extraction of astaxanthin from *Haematococcus pluvialis*. *J. Food Process. Preserv.* 40 463–472. 10.1111/jfpp.12624

